# Spatiotemporal dynamics of benthic *Microcoleus* in a Swiss lotic system

**DOI:** 10.3389/fmicb.2026.1769963

**Published:** 2026-06-09

**Authors:** Sami Zhioua, Naïma Mangia, Guillaume Cailleau, Diego Gonzalez, Pilar Junier

**Affiliations:** Laboratory of Microbiology, University of Neuchâtel, Neuchâtel, Switzerland

**Keywords:** anatoxin, benthic, communities, cyanobacteria, river, *Microcoleus*

## Abstract

Toxic species within the benthic *Microcoleus* cyanobacterial genus pose significant ecological and public health risks due to the production of the potent neurotoxin anatoxin. In Switzerland, *Microcoleus anatoxicus*, a known producer of anatoxin derivatives, has caused several dog fatalities causing alarm among the public and authorities. Similar incidents worldwide have been associated with various Oscillatoriales cyanobacteria. While previous studies have explored the microbial diversity within *Microcoleus* mats, annual spatiotemporal dynamics in lotic systems remain poorly understood. In this study, sediment samples at defined locations along the Areuse river (Neuchâtel) were collected for a year. Together with measurements of the local environmental parameters, the associated bacterial and eukaryotic communities were characterized using the 16S and 18S rRNA genes, respectively. Anatoxin-producing capacity was inferred using PCR of the *anaC* gene. The results show that while environmental parameters differed significantly over time, microbial communities differed significantly between sites. Bacterial communities showed relatively high abundances of cyanobacteria, specifically *Microcoleus*, which corresponded to up to 56% of all cyanobacteria. Furthermore, *anaC* PCR results were negative suggesting the dominance of non-toxic *Microcoleus*. Relative abundance of *Microcoleus* was highest between spring and summer, corresponding to up to 37% of the total bacterial communities. Significant positive correlations (approximately 64%) were observed between *Microcoleus* and other organisms such as rotifers, nematodes and chytrids. These positive relationships might highlight potential interactions between *Microcoleus* and predators. This is the first study of benthic microbial populations in a Swiss river. This study advances our understanding of benthic cyanobacterial ecology and provides insights into natural mechanisms that may mitigate toxin-related risks in freshwater environments. Moreover, the results highlight the prevalence of non-toxic *Microcoleus* on epilithic substrates suggesting its role in the establishment of microbial communities in the benthos.

## 1 Introduction

Cyanobacteria are phototrophic microorganisms ubiquitously found in aquatic and terrestrial environments. In aquatic ecosystems, different strains occupy defined compartments adapting their morphology and physiology to their niche (Quiblier et al., 2013). Some cyanobacteria live suspended in the water column (planktonic), while others colonize the rocky or sandy substrates of oceans, lakes, or rivers at variable depths (benthic) (Vincent, 2009). In these niches, cyanobacteria are usually present in relatively low numbers, being kept in check by limiting nutrients or by their natural enemies, which include viruses, other bacteria, and grazers (Ekvall et al., 2014; Ndlela et al., 2018; Pajdak-Stos et al., 2020; Xia et al., 2013). However, under favorable chemical and physical conditions (or when natural enemies become less efficient) excessive proliferation of cyanobacteria can follow (Biggs, 2000; Kelly et al., 2026; McAllister et al., 2018a; Paerl and Otten, 2013). For instance, elevated nitrogen and phosphorus levels or other factors such as changes in pH and temperature, can trigger an exponential increase in biomass over a short period of time (i.e., hours to days) forming a so-called cyanobacterial bloom. A subset of those blooms have a negative impact on aquatic ecosystems, constituting what is known as cyanobacterial harmful algal blooms (CyanoHABs) (Huisman et al., 2018; Paerl and Paul, 2012).

Given the diversity of habitats occupied by cyanobacteria in aquatic environments (i.e., water column or substrate-bound), the type and impact of CyanoHABs will depend on the cyanobacterial species involved (i.e., planktonic or benthic cyanobacteria) and the consequences of its uncontrolled proliferation. For instance, CyanoHABs of planktonic species result in an increase in turbidity, restricting light penetration. Moreover, the increase in suspended organic matter stimulates respiration and can cause oxygen depletion in affected ecosystems (Huisman et al., 2018; Paerl and Paul, 2012). Benthic CyanoHABs are associated with the detachment of mats during the accrual growth cycle of benthic mats (McAllister et al., 2016). The negative impact of benthic CyanoHABs is usually linked to the production of toxic peptides and alkaloids, but the production of toxins also occurs in the case of many planktonic CyanoHABs (Christensen and Khan, 2020; Huisman et al., 2018).

Benthic cyanobacteria colonize substrates such as rocks and sediments and start forming mats in association with other heterotrophic bacteria (McAllister et al., 2016). When these mats become thick enough, they can detach and float to the surface. This detachment is often due to oxygen bubbles trapped within the mat, which increase buoyancy and the likelihood of shedding (McAllister et al., 2016; Scott and Marcarelli, 2012). Benthic floating mats are notorious for the production of toxic secondary metabolites, such as microcystins or anatoxins (Huisman et al., 2018; Wood et al., 2020). These toxic floating mats can accumulate on shores due to currents, wind and waves, which, consequently, increases risk of exposure to and ingestion by domestic animals and humans (Šulčius et al., 2015). As the surface area covered by floating mats during a benthic CyanoHAB can be highly variable, the percentage of substrate coverage has been used instead to define risk scenarios and to direct response (Ministry for the Environment and Health New Zealand et al., 2024).

Dogs’ fatalities after ingesting toxic benthic cyanobacterial mats have been reported worldwide in countries such as New Zealand (Wood et al., 2007), France (Gugger et al., 2005), United States (Conklin et al., 2020; Puschner et al., 2008), Canada (Johnston et al., 2024), Mexico (Caro-Borrero et al., 2024), and Switzerland (Junier et al., 2024). More recently, toxic *Microcoleus* mats have also been reported in other countries such as Spain, although no dog fatalities have been associated to those yet (Diez-Chiappe et al., 2025). The cyanobacterial species responsible for these deaths have been identified as *Microcoleus anatoxicus* (Junier et al., 2024; Puddick et al., 2021), *Phormidium* spp. (Gugger et al., 2005; Wood et al., 2016), *Tychonema* spp. (Shams et al., 2015), and *Oscillatoria* spp. (Méjean et al., 2009). However, uncertainty in the taxonomic assignation within these groups made it difficult to assess whether these species belong to different or closely related species (Junier et al., 2024; Tee et al., 2021). Nonetheless, over the last two decades, anatoxin-producing cyanobacteria within benthic mats have consistently been assigned to the *Microcoleus* genus and these benthic proliferations have increased in frequency and geographic range around the globe (Kelly et al., 2026; Wood et al., 2020). Deaths consistently involved neurotoxic alkaloids, specifically anatoxin-a (ATX) and derivative toxins such dihydroanatoxin-a (dhATX), homoanatoxin-a, dihydrohomoanatoxin-a (James et al., 2007; Quiblier et al., 2013; Wood et al., 2020), and more recently guanitoxin (formally known as anatoxin-a(S)), which has been associated to the death of domestic animals (Fernandes et al., 2024). Genomic tools are frequently used to infer toxicity within mats (Burford et al., 2020; Christensen and Khan, 2020). For instance, qPCR targeting the *anaC* gene (Kelly et al., 2018) in the anatoxin biosynthetic gene cluster (BGC), is commonly used to evaluate the capacity of species within the mat to produce anatoxins (Junier et al., 2024; Kelly et al., 2019; Thomson-Laing et al., 2020). In addition, toxin concentration in *Microcoleus*-dominated mats might shape the associated bacteria and eukaryotes communities (Kelly et al., 2026).

To fully understand the ecological dynamics of *Microcoleus* mats, it is essential to study the associated community involving not only other bacteria but also eukaryotes. Although several studies have investigated the occurrence and dynamics of toxic benthic cyanobacteria (Bauer et al., 2023; Heath et al., 2011; McAllister et al., 2018a), few have examined the identity of other members of the microbial community within the mats (Bouma-Gregson et al., 2019; Echenique-Subiabre et al., 2018; Junier et al., 2024; Tee et al., 2020). This is an important knowledge gap, as toxic species are known to be part of complex communities that vary during mat development and act as drivers of proliferation (Bouma-Gregson et al., 2019; Thomson-Laing et al., 2020). Co-habiting taxa can modify the conditions within the mat, thus influencing the development of bloom-forming species (Kelly et al., 2026). Toxic cyanobacterial mats have been associated to heterotrophic bacteria (Berg et al., 2009; Bouma-Gregson et al., 2019; Paerl, 1996), but the associated eukaryotic communities have not been analyzed. In addition, there are only few studies on the coexistence of macroinvertebrates with benthic cyanobacteria (Caro-Borrero et al., 2024). This is a significant shortcoming given that interactions between benthic cyanobacteria and eukaryotes have also been observed. For instance, floating mats in Spain contained high abundances of juvenile and gravid female nematodes, suggesting cyanobacteria as a potential food source (Gaudes et al., 2006). Furthermore, other eukaryotic organisms including *Daphnia*, mollusks, and various zooplankton, might potentially feed on cyanobacteria (Paerl and Otten, 2013). Eukaryotic predators could be of particular interest as a potential biological mechanism to control proliferation.

The aim of this study was to determine the presence of *Microcoleus* and associated microorganisms in benthic lotic environments, and simultaneously to follow the spatiotemportal dynamics of *Microcoleus* populations during an annual cycle. We hypothesized that *Microcoleus* abundance will increase during warmer seasons, consistent with previous studies (Heath et al., 2011; McAllister et al., 2018a). Specific environmental parameters such as conductivity, temperature, or light intensity were also predicted to correlate with *Microcoleus* temporal dynamics (Biggs, 2000; McAllister et al., 2018a). In addition, PCR amplification of the *anaC* gene (Kelly et al., 2018) was used to assess the presence toxic *M. anatoxicus* in the samples. It was hypothesized that the detection of toxic strains would positively correlate with *Microcoleus* abundance. Finally, we predicted that other bacteria and eukaryotes (biological drivers of growth dynamics) would follow the same spatiotemporal patterns of *Microcoleus* abundance. In particular, we expect that the abundance of potential grazers will positively correlate with the abundance of cyanobacteria (Gaudes et al., 2006; Paerl and Otten, 2013). The data generated from this study enhances our understanding of *Microcoleus* spp. population dynamics in lotic environments and contribute to improved monitoring strategies.

## 2 Materials and methods

### 2.1 Study site and sample collection

**Figure 1:**
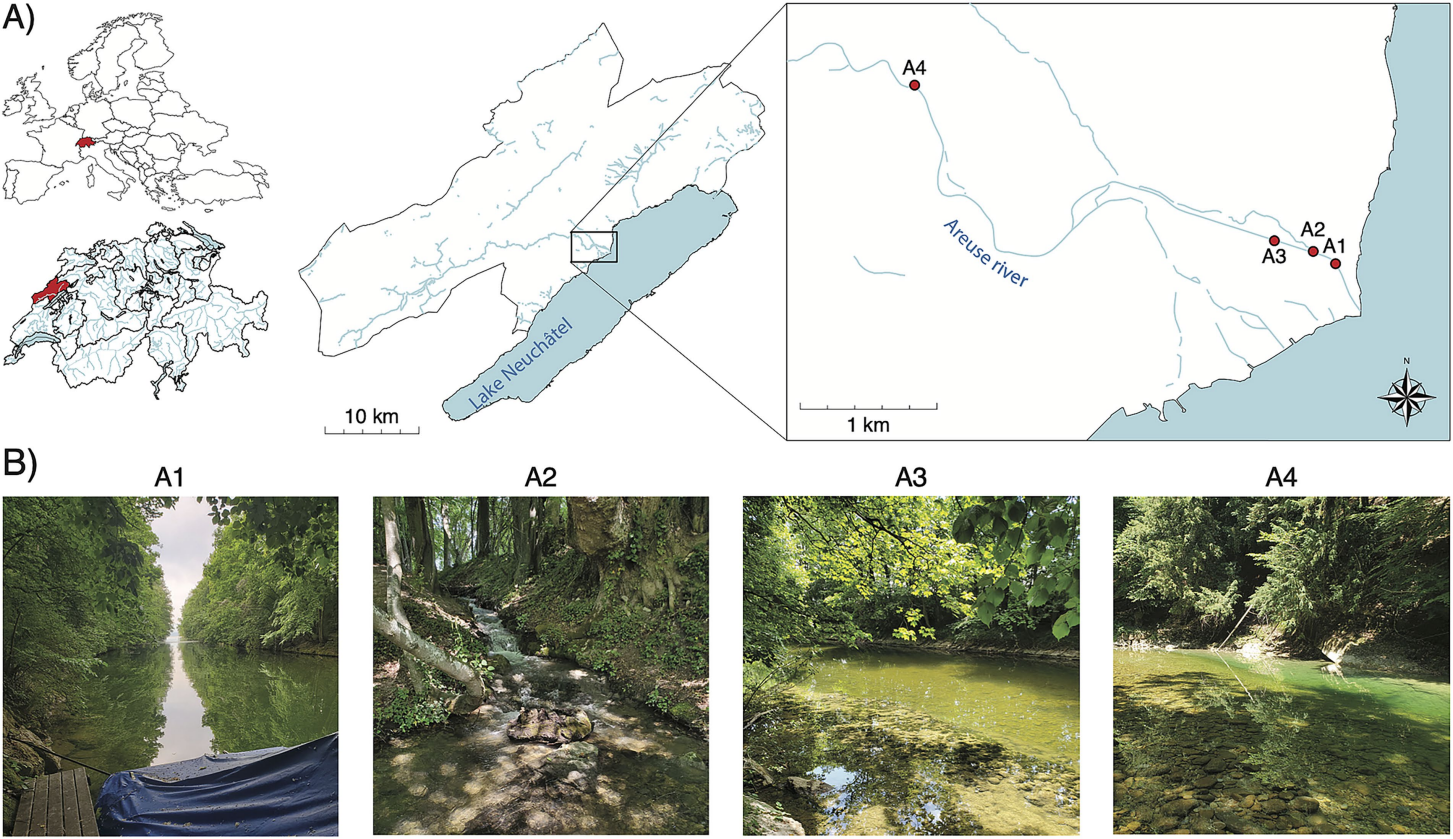
Sampling sites between the mouth and the beginning of the gorge of the Areuse river. **(A)** Map of Europe (Switzerland in red) and Switzerland, highlighting the localization of the Canton of Neuchâtel (in red). The inserts show a close-up map of the canton of Neuchâtel, Lake Neuchâtel, and the Areuse river; a zoom in the section of river indicates the locations of the four sampling sites (A1–A4). **(B)** Photos of the four sampling sites (A1–A4) are shown. The geographical location of the sites is presented in the methods.

Samples were collected monthly in the Areuse river in Neuchâtel, Switzerland between May 2022 and May 2023. The Areuse river originates in St-Sulpice (NE) at an elevation of 795 m and flows for about 32 km before reaching Lake Neuchâtel at Boudry, at an elevation of 429 m. The Areuse gorges are in the lower half of the river, between Noiraigue and Bourdy. Previous observation made by Junier et al. (2024) were done in this lower section. Based on this research, four sites were selected to analyze benthic microbial communities in the riverbed. The first site (A1) was located at the mouth of the river (46.94851321° N, 6.868667929° E, Figure 1A) where *M. anatoxicus* blooms were observed previously. The riverbank was rocky, steep and shaded. Trees on the riverbank provided shade to a large section of the mouth of the river (Figure 1B). The second site (A2) was located at the mouth of the Vivier stream (46.94932023° N, 6.866517069° E, Figure 1A). This shallow stream bifurcates from the Areuse river, crosses farmland, and enters the river again close to its mouth. This sampling site was quite flat, and the water column was very shallow (Figure 1B). The third site (A3) was located upstream (46.95002195° N, 6.862777798° E, Figure 1A) with rocky banks but the riverbed was more exposed to light (Figure 1B). Accessing site A3 was easy (when current was slow) due to a step before accessing the riverbed. The three first sites were located along the walkway to the lake where dogs are usually observed bathing in the river. Finally, the fourth site (A4) was located just downstream of the river’s gorge (46.9602384° N, 6.828133285° E, Figure 1A). Microbial benthic mats were observed at this site from the opposite bank (Figure 1B). This site is separated from the other sites by the city of Boudry. At this specific location, people and dogs are found bathing. The banks were mainly rocky and surrounded by trees, providing shade to the river. As all other sites, the riverbed was composed of mineral structures.

At each sampling site, three epilithic or sediment substrate samples were collected using an inox pot with a long handle to scrap the riverbeds’ substrate. These samples were composed of the biomass attached or over the mineral substrate (hereafter referred as samples). Samples were then placed in a 50 mL Falcon tube using metal spoons or forceps (approximately half tube-volume filled with epilithic substrate sample). In site A2, as the water column was very shallow, the benthos was scrapped using metal spoons. All sample tubes were then filled with water from the river and placed in a cooler with icepacks until being brought back to the laboratory.

An uneven number of samples was exceptionally collected at the following time points: in May 2022 at site A2 (*n* = 2) and at site A3 (*n* = 4); in July at site A3 (*n* = 4); and in October at site A4 (*n* = 0) when the river current was too strong and rendered sample collection dangerous. This resulted in a total of 39, 37, 41, and 36 samples collected from sites A1 to A4, respectively. Samples were collected at different depths between all sites: A1 = 94.2 ± 27.8 cm (mean ± standard deviation), A2 = 2.15 ± 1.73 cm, A3 = 107 ± 27.4 cm, and A4 = 109 ± 13.3 cm.

### 2.2 Environmental analyses

Physical and chemical parameters, including nutrients within the water column were measured at each site. The limit of detection from each parameter is indicated in brackets. At each sampling time, conductivity (μS/cm), dissolved oxygen (DO) (0.1–20.0 mg/L), and pH (0–14) were measured *in situ* using the HQ4300 Portable Multi-Meter with electrodes (Hach, Staad, Switzerland). Orthophosphate (0.20–4.00 mg/L PO_4_^3−^), nitrite (0.005–0.600 mg/L NO_2_^−^-N), and ammonia (0.05–1.5 mg/L NH_3_-N) concentrations were also measured *in situ* using the SL1000 Portable Parallel Analyzer ^®^ and the Chemkeys ^®^ reagents (Hach, Staad, Switzerland). For the other measurements, water samples were filtered using 0.45 μm nylon 66 syringe filters (SF2503-2, BGB ^®^, Boeckten, Switzerland). Filtered water was collected in two tubes per site (2 × 20 mL) to measure cations and anions, such as fluoride (0.2 mg/L), chloride (0.1 mg/L), nitrate (0.2 mg/L), sulfate (0.2 mg/L), sodium (0.05 mg/L), potassium (0.05 mg/L), magnesium (0.05 mg/L), and calcium (0.1 mg/L) by ion chromatography. To ensure ion stability, three drops of HNO_3_ 10% were added to the tube for measuring cations. All tubes were stored at 4 °C until further analysis. These ions were selected as those are usually analyzed as part of water quality assessments (World Health Organization, 2022). Also, 1 mL of filtered water was used for total nitrogen measurements using the LatoN Total Nitrogen (1–16 mg/L) cuvette test LCK 138 (Hach, Staad, Switzerland). Unfiltered water (50 mL) was collected in the field and frozen for urea and protein quantification to analyze different sources of nitrogen used by the benthic communities, as this has been suggested as a key factor of benthic proliferation (Kelly et al., 2026). Urea (20–50 ng/mL) was measured in the Plateforme Neuchâteloise de Chimie Analytique at the University of Neuchâtel (for the protocol, see Supplementary Information). Protein quantification was done using the Bradford assay (0.1–1.4 mg/L) following the provided protocol (B6916, Merck, Buchs, Switzerland).

Finally, three loggers (HOBO Pendant MX2202, Bourne, MA, USA) were placed in the benthos in each site. Loggers recorded light intensity (lux) and temperature (°C) between 6 a.m. and 6 p.m. during the whole sampling period. Water flow (i.e., water velocity) has not been assessed in this study because the instruments were not available during the sampling period. Nonetheless, water flow data was obtained from the hydrogeological station in the Areuse river (located between sites A3 and A4). As water flow has not been measured at each site, no correlations between water flow and the other environmental data have been analyzed.

### 2.3 DNA extraction and PCR

DNA was extracted to analyze the microbial communities. For this, approximately 500 mg of the mixed epilithic biomass-substrate sample (weighted using a precision balance) were placed into a lysis tube (using sterile tweezers) for subsequent whole DNA extraction using the MP FastDNA Spin Kit for Soil (MP Biomedicals, Santa Ana, CA, USA), following the supplier’s protocol. The amount of DNA extracted was assessed using fluorometric measurement with the Qubit ™ dsDNA Broad Range Assay Kit (Invitrogen, Carlsbad, CA, USA) and the Qubit ^®^ 2.0 Fluorometer (Invitrogen, Carlsbad, CA, USA), following the supplier’s protocol. To assess the capacity of anatoxin production, an end-point PCR of the *anaC* genes was performed as previously done without modification (Zhioua et al., 2025), targeting the *anaC* gene within *Microcoleus* species using the *Phor-AnaC-F5/R5* primers (Kelly et al., 2018). Each PCR amplification was run with a positive control corresponding to DNA from a pure culture of *M. anatoxicus* isolated from the same river (strain Neu2020) (Junier et al., 2024). The genome sequence of this strain is available under the accession number CP120853 in GenBank.

### 2.4 Community analyses

To study the composition of bacterial communities in the samples, DNA was sent directly to Fasteris SA for amplification and high-throughput sequencing. The V3-V4 regions (universal primers Bakt_341F 5-CCT ACG GGN GGC WGC AG-3′ and Bakt_805R 5′-GAC TAC HVG GGT ATC TAA TCC-3′) (Herlemann et al., 2011) were amplified with adaptors and indexes to enable sequencing on the Illumina MiSeq platform (2 paired-end 300 bp reads). Samples collected between May and August 2022, and between September 2022 and May 2023 were sequenced separately. Demultiplexed and trimmed sequences provided by Fasteris SA were analyzed using the LotuS2 pipeline (Özkurt et al., 2022). The VSEARCH (Rognes et al., 2016) algorithm was used in LotuS2 to cluster sequences into Operational Taxonomic Units (OTUs). The pipeline was modified to adjust clustering at 99% identity, otherwise the default parameters were used. OTUs were taxonomically classified using a consensus taxonomic classifier VSEARCH (Rognes et al., 2016) with the CyanoSeq (Lefler et al., 2023) and the SILVA database release 138 (Quast et al., 2012) for the 16S and 18S rRNA gene sequences, respectively. Both databases were dereplicated to the V3–V4 (16S rRNA gene) and V4 regions (18S rRNA gene) using the RESCRIPT pipeline (Robeson et al., 2021). Finally, dominant OTU sequences for the most abundant cyanobacterial genera were identified using BLAST ^®^ (Altschul et al., 1990) against the NCBI database (Sayers et al., 2022).

### 2.5 Co-occurrence network analyses

To analyze the co-occurrence patterns within the microbial communities, bacteria and eukaryote datasets were merged at the genera level. An initial filtering step was done by filtering out all OTUs with total relative abundances across all samples < 1%. Then, a Spearman correlation matrix was computed using the relative abundances of the different genera. Significant correlations (*p* < 0.05) and correlation coefficients (absolute values) > 0.6 were used in the network analyses. Detection thresholds for the OTU relative abundance were set to 1% and 2%. All networks, for all thresholds, were separated by site.

### 2.6 Statistical analysis

After measuring all environmental variables, Spearman correlations were calculated using the *rcorr* function in the Hmisc package (Harrell and Harrell, 2019). Principal Component Analysis (PCA) was done on environmental data to understand the effect of environmental parameters on the differentiation of the samples through time or space. PCA was performed with the *PCA* function in the FactoMineR package (Husson et al., 2016). Visualization of the PCA was done with the factoextra package (Kassambara and Mundt, 2017).

For community analyses, after clustering and taxonomic classification, the data was analyzed with the phyloseq package (McMurdie and Holmes, 2013). Diversity and community compositions were calculated using the vegan package (Oksanen et al., 2013). Shannon indices (*α*-diversity) were used to analyze the diversity across time, between sites, and between sequencing batches. Statistical differences were calculated using Kruskal–Wallis tests, and *post-hoc* analyses were computed using Dunn’ test. For these pairwise comparisons, *p*-values were corrected using the Benjamini-Hochberg method (Benjamini and Hochberg, 1995).

Community dissimilarity between samples (*β*-diversity) were calculated on weighted UniFrac distances. The UniFrac distance matrix was computed by aligning all OTU sequences using kalign (Lassmann, 2020), and a phylogenetic tree was built with these aligned sequences using FastTree v. 2.1.11 (Price et al., 2010). β-diversity patterns were visualized using Principal Coordinate Analysis (PCoA) using the built-in *cmdscale* function in R. Differences in community compositions were tested using a Permutational Multivariate Analysis of Variation (PERMANOVA). Homogeneity of multivariate dispersion was tested using Permutational Analysis of Multivariate Dispersions (PERMDISP). *Post-hoc* analyses were done using the Tukey’s test with similar *p*-value correction methods as described previously. Significancy levels were calculated with *p* ≤ 0.05. Analyses were all done using R (version 4.4.1).

## 3 Results

### 3.1 Environmental analyses

Environmental parameters were measured *in situ* and *ex situ* at all sites. Several parameters were excluded from the analysis because the values obtained were always under the limit of detection. This was the case for fluoride, urea, and proteins. Several environmental parameters shown a sharp increase in concentrations at the beginning of the sampling period, plateaued, and decrease slightly at the beginning of winter (Nov-Dec). This was the case for calcium, conductivity, nitrate, DO, pH, and total nitrogen. An opposite trend was observed in chloride, magnesium, nitrite, sodium, sulfate and temperature. Light intensity and ammonia do not show such trends and less variable over the sampling period (Supplementary Figure S1).

Calcium and sulfate correlated with all other variables except for nitrite and phosphate, respectively. Other environmental factors showed significant correlations, but the coefficients were relatively low (Supplementary Figure S2). Light intensity *in situ* also showed significant correlations with conductivity, nitrate, and calcium, but the correlation coefficients did not exceed 0.5. Total nitrogen and different forms of nitrogen were also measured. Results suggested a strong positive correlation between total nitrogen and nitrate (Spearman correlation coefficient = 0.90, *p* < 0.001, Supplementary Figure S2). Furthermore, nitrate concentrations were the highest recorded, thus accounting for almost all nitrogen within the samples.

A Principal Component Analysis (PCA) on environmental data was carried out to understand the effect of environmental parameters on the differentiation of the samples through time or space. This analysis showed several factors that correlated significantly to the two dimensions. The first PC (explaining 52.4% of the variance) was driven by temperature (92.6%), calcium (88.9%), sulfate (83.4%), DO (79.5%), and nitrate (76.9%). The second PC (explaining 9.7% of the variance) was driven by pH (73.8%), total nitrogen (49.1%), and light intensity (34.7%). The quality of representation (cos^2^) of all variables on both dimensions is illustrated in Supplementary Figure S3A.

The initial PCA was simplified by selecting top four variables that correlated best with both dimensions (correlation), and that showed high quality of representation by the dimension (cos^2^). The quality of representation of the variables on each axis was considered, retaining only those with cos^2^ > 0.2. As nitrate dominated the nitrogen within the samples, only this form of nitrogen was accounted for in the variable rarefaction. This second PCA resulted in temperature strongly correlating to the first PC (96%), along with calcium (90.9%), DO (89.3%), nitrate (88.7%), and sulfate (85.1%). This dimension explained 62.5% of the variation. The second PC correlated with pH (71.3%) and light intensity (60.5%) while explaining 16.2% of the variation. The quality of representation (cos^2^) of all variables on both dimensions is illustrated in Supplementary Figure S3B.

**Figure 2:**
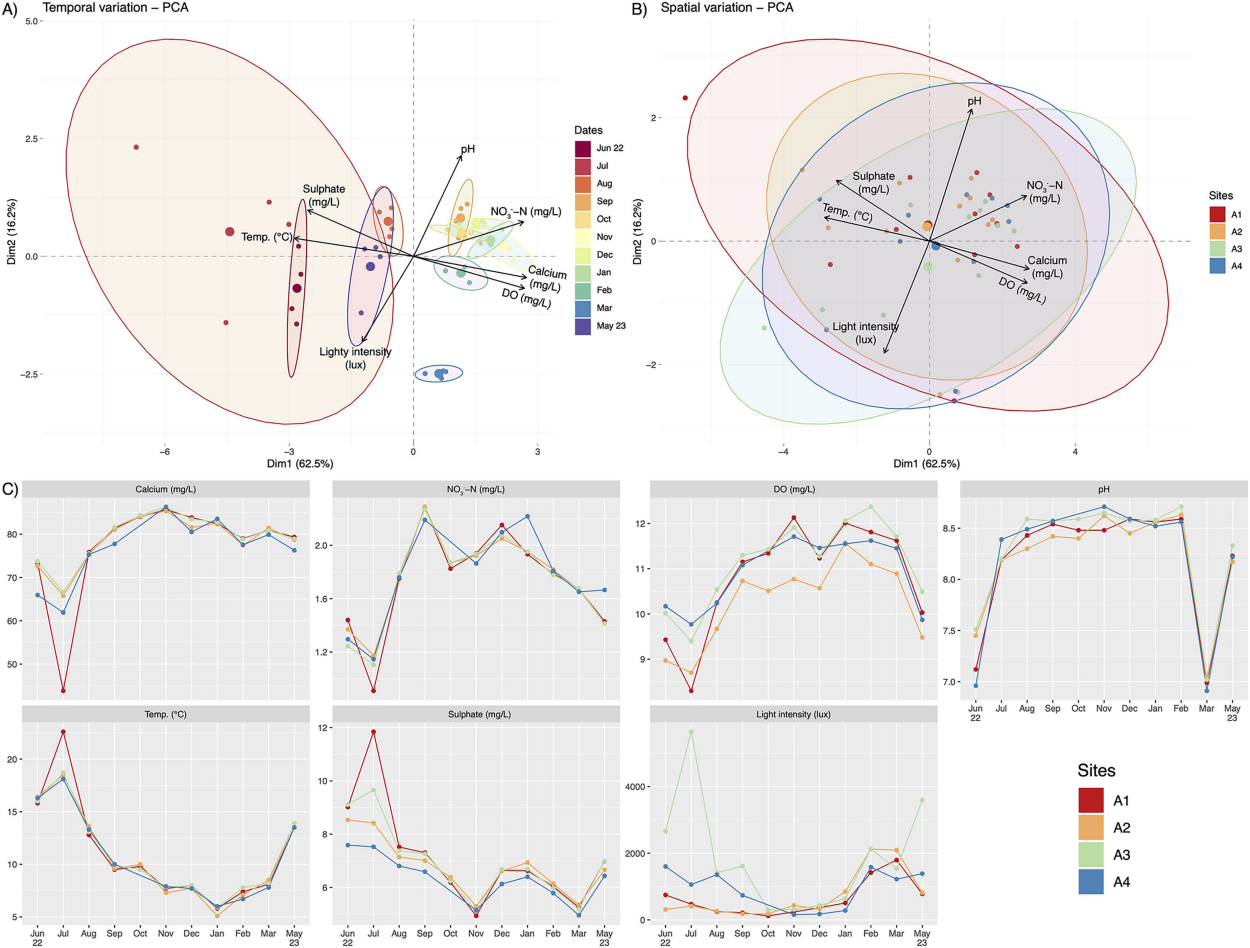
Environmental analysis of the selected parameters after simplification. **(A,B)** PCA of the selected parameters grouped and colored by time **(A)** and by site **(B)**. Larger points are the centroids, and smaller points are the environmental sample. Ellipses represent the confidence intervals at 95%. Differences throughout time are visible compared to overlapped ellipses and centroids between sites. **(C)** Time-variation of the selected parameters throughout the sampling period. Parameters and units are described above each panel.

PCA suggested that samples differ throughout time rather than between sites (Figures 2A,B). Indeed, environmental factors varied seasonally across the river, but this applies to all the sites. Calcium, nitrate, DO, and pH all showed a drop in July before increasing until January. Water temperature and sulfate showed opposite trends. Between all factors, site A1 shows the highest/lowest peaks. Regarding light intensity, sites A1 and A2 showed lower light intensity compared to sites A3 and A4. From January, light intensity increased across all sites, with site A3 showing the highest intensities (Figure 2C). Our observations suggest that both sites A1 and A2 are the most covered by trees, compared to A3 and A4 where the sampling sites are more exposed to sunlight (Figure 1B). Our analyses suggested that environmental conditions remained similar between all sites but showed seasonal dynamics.

### 3.2 Microbial community analysis

To analyze the benthic microbial communities for all 154 samples, we sequenced the V3-V4 and the V4 regions within the 16S and 18S rRNA genes, respectively. Regarding the bacterial communities, a total of 20′839 OTUs were obtained across all four sites. These OTUs were classified into 52 phyla, 125 classes, 258 orders, 473 families, and 847 genera. Cyanobacteria were categorized into 3 classes, 20 orders, 37 families, and 63 genera. Eukaryotic sequences represented 10′045 OTUs. These OTUs were classified into 72 phyla, 195 classes, 390 orders, 520 families, and 879 genera. Further details regarding the different phyla are provided in the Supplementary Tables S1, S2 (bacteria and eukaryotes, respectively). OTUs and the number of reads per sample are provided in Supplementary Table S3.

#### 3.2.1 Diversity of microbial communities

**Figure 3:**
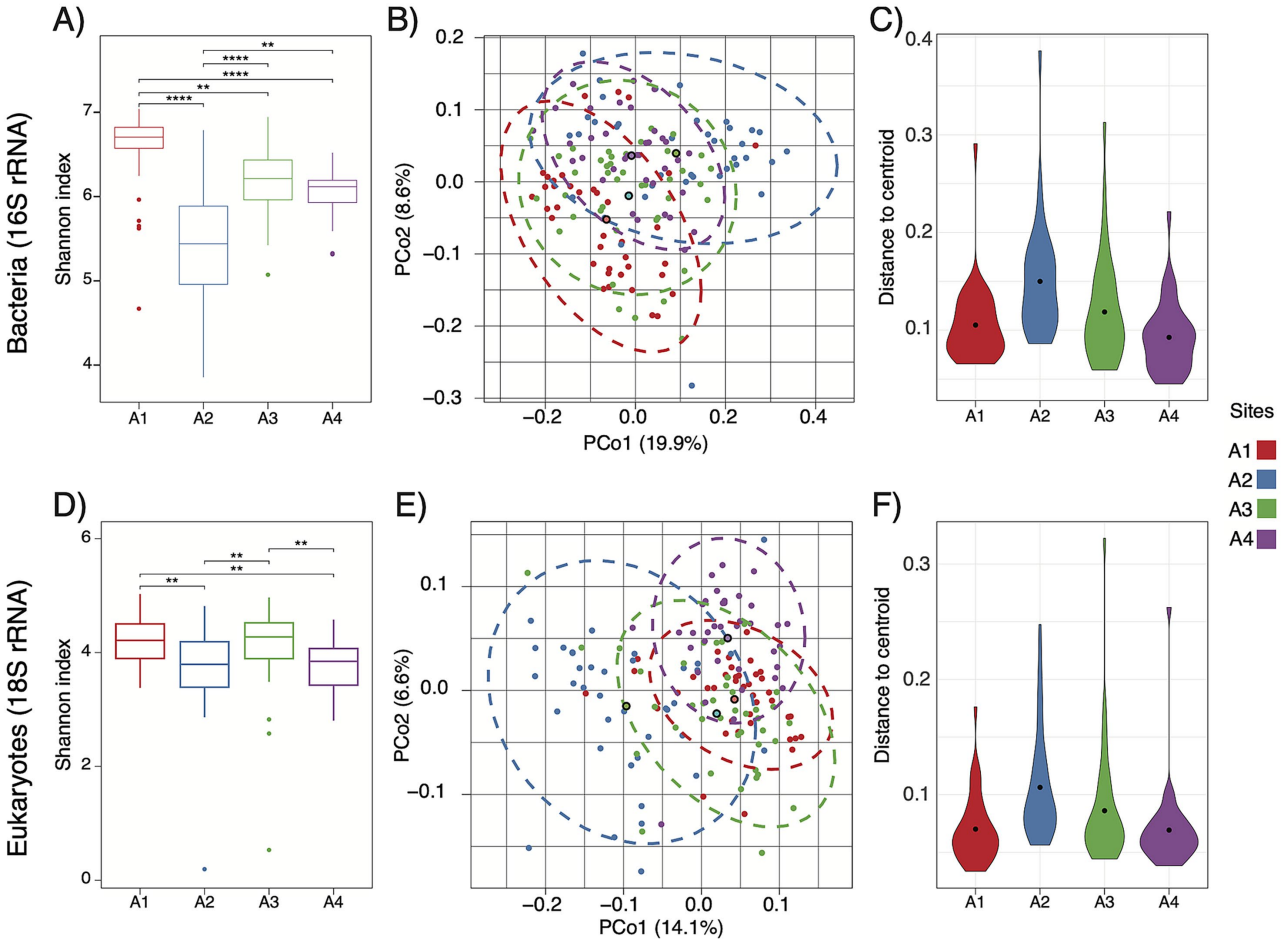
Microbial community differences between sites (differentiated by colors). **(A–D)** *α*-Diversity of the bacterial and eukaryotic communities calculated with the Shannon index. Boxplots illustrate the interquartile range (IQR); middle line represent the median; the whiskers represent the most extreme data points within 1.5 times the IQR; and the points represent the outliers. Significancy codes: **** < 0.0001, *** < 0.001, ** < 0.01, * < 0.05. **(B,E)** *β*-Diversity: PCoA of the bacterial and eukaryotic communities calculated using a UniFrac distance metric, respectively. Points represent the samples and dashed ellipses (confidence intervals at 95%). Points with black borders represent the centroids. **(C,F)** Multivariate dispersions across all sites. Black points represent the means between each site.

Differences in bacterial diversity within samples (*α*-diversity) were evaluated comparing the Shannon index. Bacterial α-diversity was significantly different among site (Kruskal-Wallis Rank Sum Test: *χ*^2^ = 69.078, df = 3, *p* < 0.001), with post-hoc Dunn tests indicating that all pairs were significantly different except between A3 and A4 (Figure 3A). High α-diversity values were observed across all sites, with the highest index calculated in site A1 (median = 6.73), and the lowest in site A2 (median = 5.45). However, Shannon diversity did not differ significantly between sequencing batches (Kruskal–Wallis Rank Sum Test: *χ*^2^ = 1.386, df = 1, *p* = 0.2391, Supplementary Figure S4A) or across time (Kruskal–Wallis Rank Sum Test: *χ*^2^ = 15.325, df = 12, *p* = 0.2241, Supplementary Figure S5A). These findings illustrate spatial variation of bacterial α-diversity within samples rather than a temporal effect.

Differences in community compositions (*β*-diversity) were quantified using a UniFrac distance matrix and visualized using PCoA. Our results revealed a significant spatial effect on bacterial communities’ compositions (PERMANOVA: *F* = 10.863, *p* < 0.001, Figure 3B). *Post-hoc* Tukey’s test revealed significant differences between the following community pairs: A1–A2, A2–A3, and A2–A4 (Supplementary Table S4). Multivariate dispersion analysis also revealed significant variability among sites (PERMDISP: *F* = 10.193, *p* < 0.001, Figure 3C), indicating strong compositional heterogeneity across sites. On the other hand, dispersion was not affected by the sequencing batch (PERMDISP: *F* = 0.128, *p* = 0.739, Supplementary Figure S4C) or by time (PERMDISP: *F* = 0.929, *p* = 0.527, Supplementary Figure S5C). Although, analyses did indicate a significant overall effect of sequencing batches (PERMANOVA: *F* = 3.867, *p* = 0.0052, Supplementary Figure S4B) and of time (PERMANOVA: *F* = 3.672, *p* < 0.001, Supplementary Figure S5B) on bacterial communities. As dispersion remained homogenous across time and sequencing batches, spatial factor was the dominant driver of β-diversity bacterial patterns.

Eukaryotic α-diversity was also significantly affected by site (Kruskal-Wallis Rank Sum Test: *χ*^2^ = 24.31, df = 3, *p* < 0.001, Figure 3D), with significant pairwise differences across all comparisons except for A1–A3 and A2–A4 (Figure 3D). In contrast, Shannon diversity was not significantly different between sequencing batches (Kruskal–Wallis Rank Sum Test: χ^2^ = 0.587, df = 1, *p* = 0.4436, Supplementary Figure S4D) or across time (Kruskal–Wallis Rank Sum Test: χ^2^ = 18.142, df = 12, *p* = 0.114, Supplementary Figure S5D). These similar patterns observed with the bacterial diversity illustrate a strong spatial effect compared to other factors.

Eukaryotic β-diversity showed similar patterns as the bacterial β-diversity with a significant effect of site between communities’ composition (PERMANOVA: *F* = 13.559, *p* < 0.001, Figure 3E). *Post-hoc* Tukey’s test indicated significant differences between sites A1–A2 and A2–A4 (Supplementary Table S4). PERMDISP also revealed significant variability between sites (*F* = 6.645, *p* < 0.001, Figure 3F). However, dispersion was not affected by time (PERMDISP: *F* = 0.612, *p* = 0.83, Supplementary Figure S5F) or sequencing batch (PERMDISP: *F* = 0.639, *p* = 0.422, Supplementary Figure S4F). PERMANOVA also detect a significant effect of time (*F* = 2.025, *p* < 0.001, Supplementary Figure S5E) and sequencing batches (*F* = 3.206, *p* = 0.0037, Supplementary Figure S4E). As for bacterial β-diversity, site-factor was the most impactful driver on eukaryotic β-diversity compared to time and sequencing batch.

#### 3.2.2 Microbial diversity and community composition

Bacterial communities were predominantly composed of Proteobacteria (annual mean (*μ*) = 37.3%) and Cyanobacteria (*μ* = 28%), which together constituted approximately 60–70% of the total bacterial communities, year-round and across all sites. Uncultured Bacteria (*μ* = 3.7%) and other phyla, such as Planctomycetes (*μ* = 7.3%) and Actinobacteria (*μ* = 6.5%) were present but at much lower abundances. Cyanobacteria generally showed a decline during summer before increasing in abundance during winter and early spring, often mirroring the dynamics of Proteobacteria (Supplementary Figure S6). Cyanobacteria averaged between 18% and 39% of the bacterial communities’ relative abundance (Table 1). Maximum relative abundances were reached between March and June corresponding approximately to 35% to 56% of the whole bacterial community (Table 1).

**Table 1:** Summary of the phylum cyanobacteria and genus *Microcoleus* relative abundance (percentage; %) in each site.

Site	Cyanobacteria		*Microcoleus*		
	Annual mean abundance (%)	Max. abundance (%)	^a^Annual mean abundance (%)	^a^Max. abundance (%)	^b^Max. abundance (%)
A1	18.3 ± 9.4	June: 35	37.8 ± 9.8	May: 56.2	June: 20.9
A2	38.2 ± 14.1	April: 56	45.8 ± 12.3	July: 63.9	April: 36.5
A3	25.3 ± 10.6	March: 40	25.5 ± 11.6	April: 52.8	April: 16.2
A4	30.2 ± 5.8	May: 37	35.9 ± 5.8	July: 50.6	July: 17.1

Annual mean abundances are represented by the mean ± the standard deviation. Maximum relative abundances are described as the month and the proportion. Statistics for the phylum cyanobacteria are described based on all bacterial phyla. *Microcoleus* statistics are calculated ^a^ only within the cyanobacterial communities, and ^b^ within all bacterial communities.

**Figure 4:**
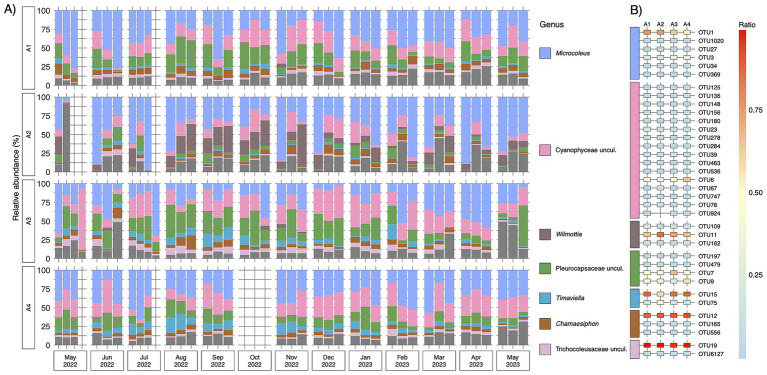
Temporal and spatial dynamic of the top 5 genera of cyanobacteria across all sites. **(A)** Total relative abundance (%) of these genera among cyanobacteria in each site (*y*-axis). The temporal scale is given on the *x*-axis. Each genus is separated by color. All other genera that are not described are included in others (grey bars). Empty bars are samples that could not be collected due to weather conditions. **(B)** Heatmap of the ratio of each OTU assigned to the genus per site. OTUs of the genera are grouped together. A colored scalebar ranges from blue to red, illustrating the lower to higher ratios of OTU within the genus.

Cyanobacteria populations were dominated by the genus *Microcoleus*. The relative abundance of *Microcoleus* averaged between 26 and 46% of the cyanobacterial communities. Maximum prevalence ranged between 51% to 64% of the total cyanobacterial community during end of spring to summer (Figure 4; Table 1). Moreover, *Microcoleus* reached 16% to 37% of the total bacterial communities during the same seasons (Table 1). The genus *Microcoleus* was dominated by OTU 1 (Figure 4). The top BLAST hits of this OTU were sequences of different Oscillatoriales including *M. anatoxicus* with 99% similarity over 408 bps (Accession n° MW405023.1). Other dominant OTUs (OTU 3 and OTU 27) showed maximum relative abundances > 20% (Figure 4) and included *M. anatoxicus* with 99% similarity as top BLAST hits. Surprisingly, all samples were negative for the presence of the *anaC* gene, which suggested that the *Microcoleus* species detected were not anatoxin producers.

Other dominant groups of cyanobacteria were observed at high relative abundance. The group assigned to uncultured Cyanophyceae was the second most dominant group. Two OTUs were dominant within this group (maximum relative abundances > 20%): OTU 6 and OTU 180 (Figure 4). The BLAST hits for OTU 6 showed sequences of Pseudanabaenaceae with 99% identity over 402 bps (Accession No. PP490678.1). The BLAST hits for OTU 180 also showed sequences of *Pseudanabaena* sp. with 92% identity over 405 bps (Accession No. FJ712266.1). Seasonal dynamics of these cyanobacteria showed opposite trends to those of *Microcoleus* in sites A1, A3, and A4.

Site A1 also showed relatively high abundance of uncultured Pleurocapsaceae with rates increasing until end of autumn in sites A1 and A3. The top BLAST hit for dominant OTUs (OTU 7 and OTU 9; maximum relative abundances > 20%) were two sequences of *Pleurocapsa* sp. with 99% of identity over 408 bps (Accession No. KC525081.1) and 96% of identity over 405 bps (Accession No. OY762512.1), respectively (Figure 4).

Site A2 showed high relative abundance of a group assigned to the *Wilmottia* genus. The OTU that dominated this genus was OTU 11 (maximum relative abundances > 20%), which had the most recurrent BLAST hits for sequences of *Wilmottia murrayi* with approximately 98–99% identity over 405 bps. Seasonal dynamics of *W. murrayi* appeared to follow opposite trends to those of *Microcoleus* sp. (Figure 4).

**Figure 5:**
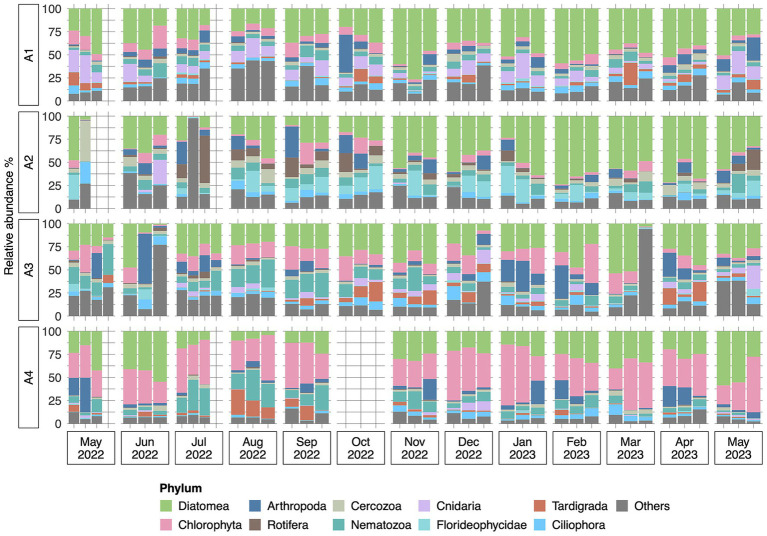
Temporal and spatial dynamic of the relative abundance of the top 5 phyla of eukaryotes across all sites (*y*-axis). The temporal scale is given on the *x*-axis. Each phylum is separated by color. All other phyla that are not described are included in others (grey bars). Empty bars are samples that could not be collected due to weather conditions.

Eukaryotic communities were also very diverse with samples dominated by several phyla (Figure 5). Samples contained mainly diatoms (*μ* = 33.3%) and chlorophytes (*μ* = 15.5%), but other phyla were also highly present such as nematodes (*μ* = 8.5%) and arthropods (*μ* = 7.2%) (Figure 5). Among these phyla, different genera were abundant (Supplementary Figure S7). For instance, in July at site A2, maximum relative abundances of Bdelloidea rotifers reached 49%. Chromadorea and Monhysterida nematodes reached maximum relative abundance of > 20% in samples collected in July at site A4 and in May at site A3, respectively (Supplementary Figure S7). *Navicula* diatoms showed maximum relative abundances of 45%. Furthermore, chlorophytes were dominated by the Ulvophyceae, reaching up to 50% of the total eukaryotic community. These results suggest relatively high abundances of photosynthetic organisms but also zooplanktonic microorganisms that could potentially graze on cyanobacteria.

#### 3.2.3 Co-occurrence network analyses

**Figure 6:**
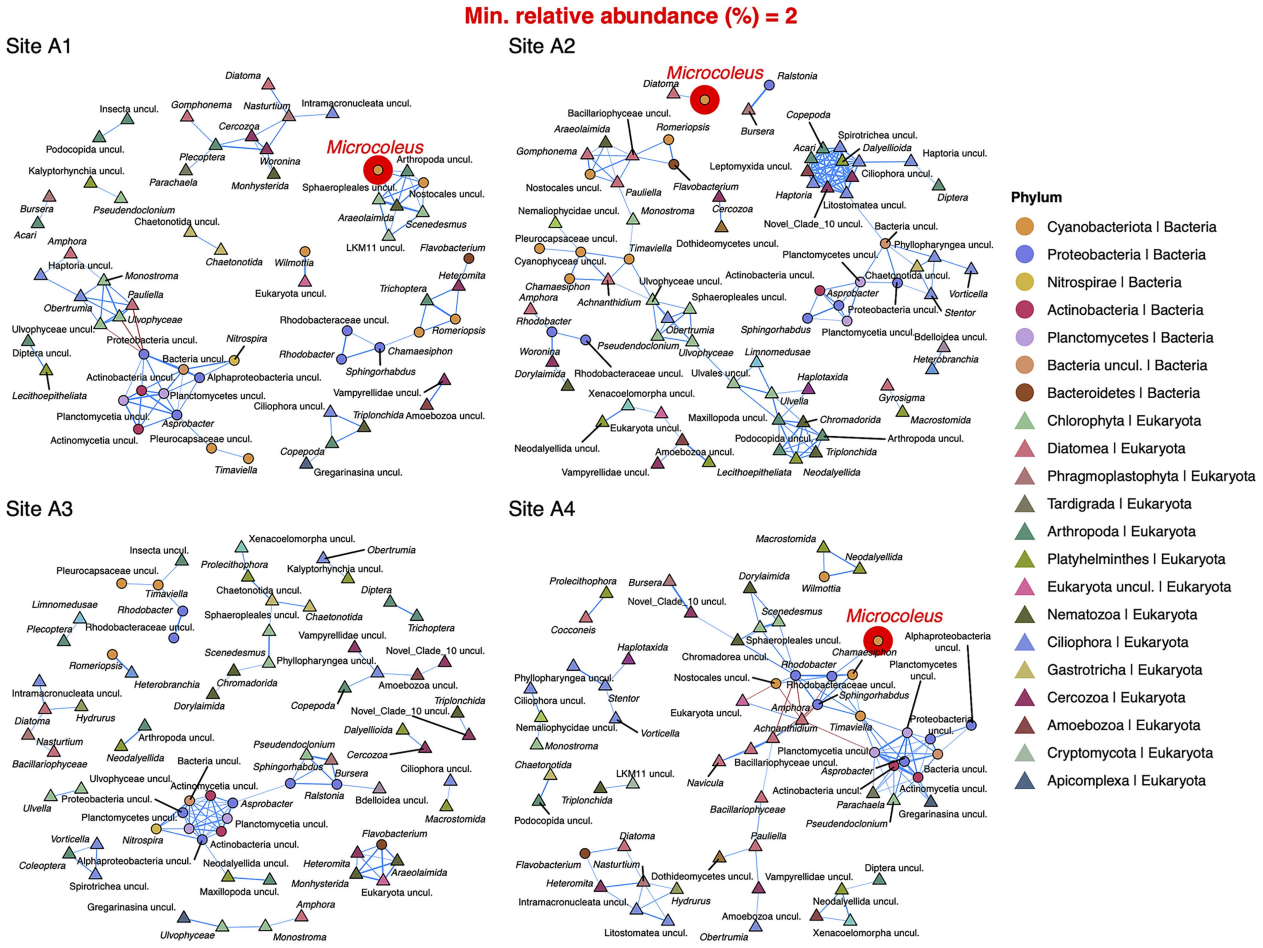
Co-occurrence network analyses of the microbial communities between each site (minimum relative abundance at 2%). Positive and negative correlations are colored in blue and red, respectively. Correlation strengthens is illustrated by the thickness of the correlation vertices (correlations illustrated: *p* < 0.001 and *r* > 0.6). Nodes are colored by phylum. Bacteria and eukaryotes are differentiated by round and triangle nodes, respectively. No correlations were observed with *Microcoleus* in site A3.

To better understand interspecific interactions, we investigated the co-occurrence between *Microcoleus* and other microorganisms. For this, network analyses were performed based on the relative abundance (≥2%) of the different bacteria and eukaryote genera combined. The total number of edges and nodes in our simplified co-occurrence network analyses ranged from 86 to 140 and from 60 to 73, respectively. More specifically, the number of edges and nodes per site are described as following: site A1 = 90 and 60; site A2 = 140 and 73; site A3 = 86 and 69; site A4 = 105 and 61 (edges and nodes, respectively, Figure 6). Our network analysis also illustrated 23 shared edges between sites A3-A4 (13 edges) and between A2-A3-A4 (10 edges). No edges were shared with site A1. Common edges are described in Supplementary Table S5.

Co-occurrences between *Microcoleus* and other species were observed in sites A1, A2 and A4. Interestingly, these co-occurrences with *Microcoleus* were unique to each site and were always positive. In site A1, *Microcoleus* showed positive correlations with Nostocales cyanobacteria (70.8%), with Sphaeropleales algae (74%), with Araeolaimida nematodes (80.5%) and with arthropods (62.3%). In site A2, *Microcoleus* showed correlations with diatoms (63.5%). Finally, in site A4, *Microcoleus* was positively correlated with Rhodobacteraceae (68.1%) (Figure 6; Supplementary Table S5).

A second network was constructed lowering the threshold of relative abundance to ≥ 1%. This revealed additional correlations between *Microcoleus* and other microorganisms. In site A1, *Microcoleus* was positively correlated with Trichocoeusaceae (75.8%) and *Apatinema* cyanobacteria (81.6%), and other eukaryotes such as Chlorophytes (64.2%), Laboulbeniomycetes fungi (83.8%), *Centramoebida* amibeae (82.9%), *Adinetida* rotifers (63.4%) or Monhysterida nematodes (64%). Furthermore, positive correlations appeared in site A2 with *Luteolibacter* (60.7%) and uncultured Rhizophydiales (63.4%). In site A4 no additional correlations were observed (Supplementary Figure S8; Supplementary Table S6).

## 4 Discussion

### 4.1 Environmental variables

In this study, samples from benthic substrates were collected, and various environmental parameters and microbial communities were analyzed to better understand the ecology of benthic microorganisms in the Areuse river, with emphasis on *Microcoleus* spp. The analysis of environmental parameters indicated that seasonal variation was more relevant than spatial dynamics and similar trends in water temperature and nutrient loads have been reported in other Swiss rivers (Michel et al., 2020; Zobrist et al., 2018) and across other European rivers (Di Nunno et al., 2024; Schauer et al., 2025). In contrast, the significant differences of microbial communities between sampling sites indicate that other environmental factors such as sediment composition and flow (Wilkes et al., 2019; Wohl et al., 2015) might be more relevant in habitat structure. Indeed, water flow has a significant impact on rivers’ chemistry. Low water discharge can result in a nutrient increase consequently driving eutrophication and acidification. Furthermore, high water flow will increase organic matter and sediment influx, which can reduce pH (Nilsson and Renöfält, 2008). Data provided by a hydrogeological station showed variations of water flow in the Areuse river (Supplementary Figure S9). These dynamics followed similar seasonal patterns of calcium, nitrate, DO, and pH, suggesting a potential impact of water flow on these parameters.

### 4.2 Benthic microbial communities

The benthic community composition was significantly affected by site, time, and sequencing batch. While time and sequencing batch did not alter the variability (dispersion) within groups, the observed microbial communities were mainly driven by site. Indeed, *Microcoleus*-dominated mats have shown similar patterns in which microbial communities were also driven by location (Echenique-Subiabre et al., 2018; Thomson-Laing et al., 2020). The dynamics of bacterial communities along the Areuse river showed that cyanobacteria were largely present across the samples, with *Microcoleus* dominating the cyanobacterial communities. The genus *Microcoleus* includes species that are reported to be the most prominent and ubiquitous group of cyanobacteria, inhabiting both terrestrial (Stanojković et al., 2024) and benthic riverine environments (McAllister et al., 2016). In biological soil crusts or arid and semiarid ecosystems, the non-toxic *M. vaginatus* was identified as a keystone species that impacts features such as soil stability, primary production, and carbon influx (Stanojković et al., 2024; Xiao et al., 2023). The detection of putative non-toxic *Microcoleus* as the dominant member of cyanobacterial communities in the benthic samples might indicate a similar role for epilithic communities, but this needs to be verified in future studies.

The sequencing method used here (i.e., short-read sequences) allowed a limited taxonomic resolution. When aligning OTUs assigned to *Microcoleus* against the NCBI database, OTU 1 showed 99% identity with *M. anatoxicus*, a known producer of ATX and dhATX (Conklin et al., 2020; Junier et al., 2024). However, amplification of the *anaC* gene from the anatoxin BGC was negative for all the samples. This suggests that the *Microcoleus* strains present in the benthic samples are likely non-toxic and that *M. anatoxicus* is absent or poorly represented, despite the fact that it was previously reported in floating mats and benthic communities in the same river (Junier et al., 2024). Co-occurrence of toxic and non-toxic *Microcoleus* strains has been reported in dense mats (Junier et al., 2024; Tee et al., 2021; Valadez-Cano et al., 2023). One hypothesis that might explain the absence of *M. anatoxicus* in the samples could be that that toxic *Microcoleus* might be better adapted to dense mats rather than sparse communities within epilithic substrate samples. Previous research has specifically focused on cyanobacterial mats in rivers (Bouma-Gregson et al., 2019; Johnston et al., 2024; Junier et al., 2024; Kelly et al., 2019; Valadez-Cano et al., 2023). Those studies reported that *Microcoleus* and their toxins were highly abundant during summer, as these months were warmer and discharge was stable (Heath et al., 2011). In this study, samples were systematically scrapped at defined sites rather than focusing on the collection of visible cyanobacterial mats. This type of sampling likely corresponds to a different habitat within the benthos, but the results still highlight the importance of *Microcoleus* as a founding member of epilithic benthic communities, particularly during the summer. Other groups of cyanobacteria with high relative abundances such as *Pleurocapsa* sp., *Pseudanabaena* spp. or *Wilmottia murrayi*, are all commonly found in rivers (Giblin and Gerrish, 2020; Lee et al., 2019; Loza et al., 2013; Taş et al., 2019). *Pseudanabaena* appeared to co-occur with other cyanobacteria such as *Planktothrix* (Giblin and Gerrish, 2020), or in arctic benthic mats was associated with Oscillatoriales cyanobacteria (Zhang et al., 2015), however these interactions have not been observed in our analyzes.

Previous studies have explored interactions between *Microcoleus* and other members of the microbial community. For instance, non-toxic *Microcoleus* mats showed higher abundances of Burkholderiales than toxic *Microcoleus* mats (Bouma-Gregson et al., 2019; Wood et al., 2020). These heterotrophic bacteria were also observed here in all samples at lower abundances compared to cyanobacteria. In the Areuse river, *M. anatoxicus* has showed positive and negative correlations with *Pedobacter* sp. and *Gemmatimonas* sp., respectively (Junier et al., 2024). These interactions were not observed here in a general co-occurrence network analysis focusing on *Microcoleus*. In addition, those interactions were observed within floating toxic mats while this study focused on epilithic sediments. Regarding interactions with eukaryotic species, *Microcoleus* was positively correlated with nematodes, rotifers or parasitic chytrids (i.e., Rhizophydiales). Araeolaimida and Monhysterida nematodes have been reported free leaving in freshwater globally (Traunspurger, 2014). Nematodes have been observed abundantly in cyanobacterial biofilms, suggesting cyanobacteria as a potential food source. In addition, nematode density was positively correlated with cyanobacterial biomass (Gaudes et al., 2006; Majdi et al., 2011). These nematodes were identified as epistrate feeders, which have a small tooth enabling them to feed on algae (Traunspurger, 2014). The positive correlation with nematodes in the co-occurrence networks could guide specific strategies to enrich those from benthic samples in the future. Rotifers, especially the genus *Adinetida,* have also been described in cyanobacterial mats (Lukashanets and Maisak, 2023; Tee et al., 2020; Velázquez et al., 2017; Wada et al., 2023). The same genus was observed in the epilithic benthic mats in the Areuse river. Like nematodes, they are reported to feed on cyanobacteria, specifically in river biofilms (Mialet et al., 2013; Velázquez et al., 2017). Network analyses also showed significant positive correlations between *Microcoleus* and chytrids. Chytrids are aquatic parasitic fungi that infect phytoplankton including filamentous cyanobacteria such as *Anabaena* or *Planktothrix* (Frenken et al., 2020; Gerphagnon et al., 2013; McKindles et al., 2021). Furthermore, chytrids might indirectly affect the abundance of cyanobacteria by fragmenting the filaments, thus facilitating grazing of other zooplanktonic organisms (Frenken et al., 2020). These different interactions highlight the importance of studying the whole microbial communities by underlining potential trophic interactions.

This research highlighted the annual dominance of non-toxic *Microcoleus* in epilithic sediments along the Areuse river. In addition, *Microcoleus* positively correlated with other microorganisms such as parasitic chytrids or potential grazers, highlighting trophic interactions within these sediments. Despite covering gaps from previous research, spatiotemporal dynamics of microbial populations in rivers should include other environmental parameters. River flow and sediment composition, which have not been measured in this study, are also expected to contribute to *Microcoleus* ecology. Indeed, water flow has been reported to alter the relative abundance and proliferation of toxic *Microcoleus* (Heath et al., 2011; Johnston et al., 2024; McAllister et al., 2018b, 2016; Wood et al., 2017), which might lead to seasonal changes in toxin concentration. Furthermore, nutrients and minerals are transported within the river and can accumulate into the sediments. Inputs and outputs of these sediments could indirectly affect the habitat structure within the river (Wohl et al., 2015). Stabilization of sediments by extracellular polymeric substances (EPS) produced by the resident microbiota in the mats could result in a higher nutritional input of these sediments (Vincent, 2009; Wilkes et al., 2019).

While this study advanced our understanding of benthic communities (particularly within the Areuse river), there are aspects that require further investigation. For instance, in the future benthic mats should be targeted to analyse the ratio of toxic and non-toxic *Microcoleus* and to correlate this with toxin concentrations. This would enhance our understanding of the interplay between toxic and non-toxic strains on the development of toxic mats. Another important aspect to consider is the effect of temporal dynamics in river flow over the development of benthic communities. As indicated previously, water discharge can not only affect other environmental parameters such as pH or nutrient content (Nilsson and Renöfält, 2008) but also the abundance of toxic strains and consequently toxin concentrations (Heath et al., 2011; Johnston et al., 2024; McAllister et al., 2018b, 2016; Wood et al., 2017). Together, these studies would significantly improve our knowledge of benthic mats within Swiss rivers and similar lotic environments.

## Data Availability

The raw sequencing data generated in this study have been deposited in the BioProject database at the National Center for Biotechnology Information (NCBI) under accession number PRJNA962543.
