# Supplementary material for: Spatiotemporal dynamics of benthic Microcoleus in a Swiss lotic system
Source: Front Microbiol. 2026 Jun 9;17:1769963. doi: 10.3389/fmicb.2026.1769963 (PMC13287139; doi:10.3389/fmicb.2026.1769963)
Supplement: Supplementary file 1 [file Data_Sheet_1.PDF]

Supplementary figures

Fig. S1: Time-variation of all environmental parameters throughout the sampling period. Parameters and units are described above each panel. Each environmental parameter is differentiated by site (x-axis).

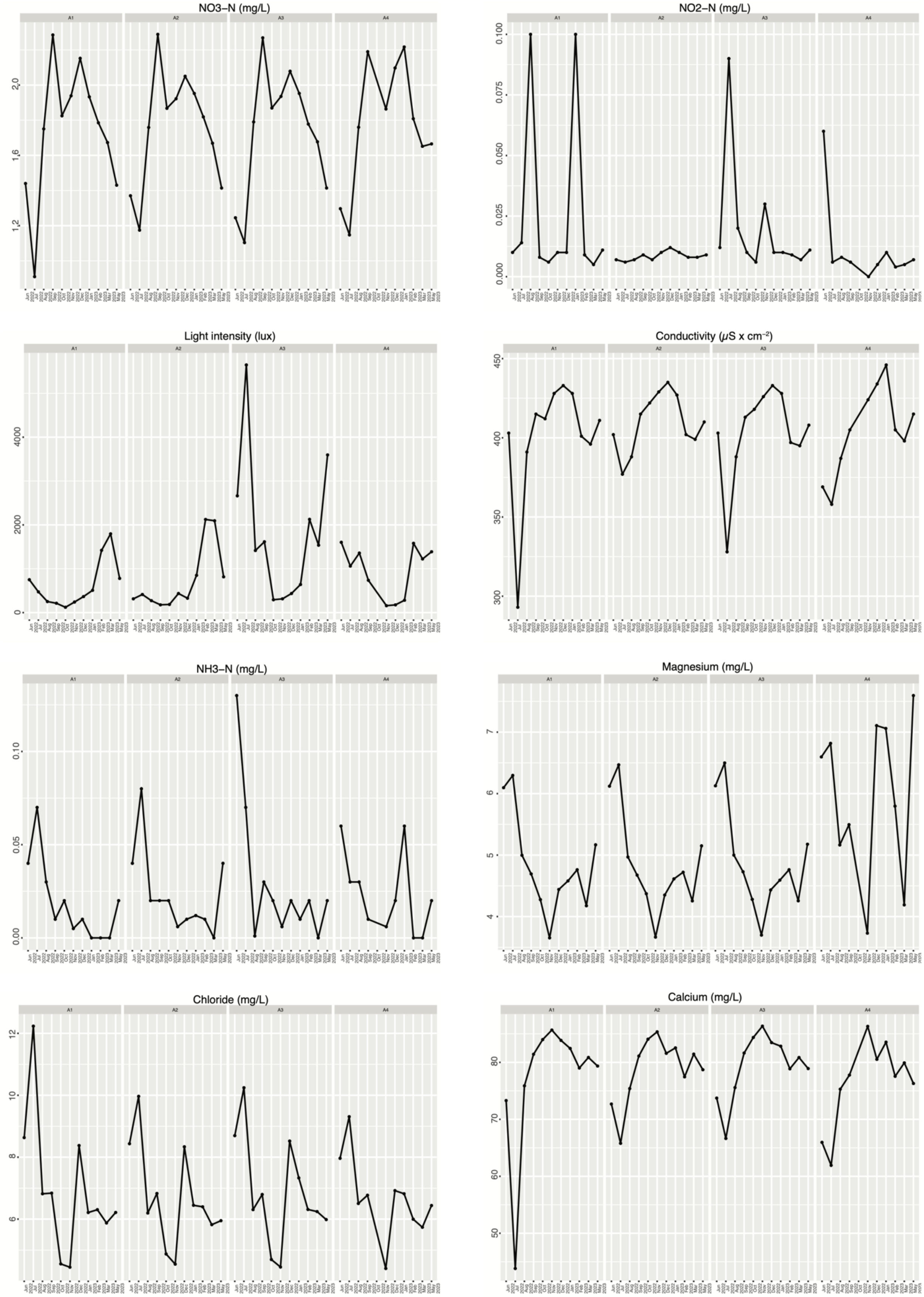

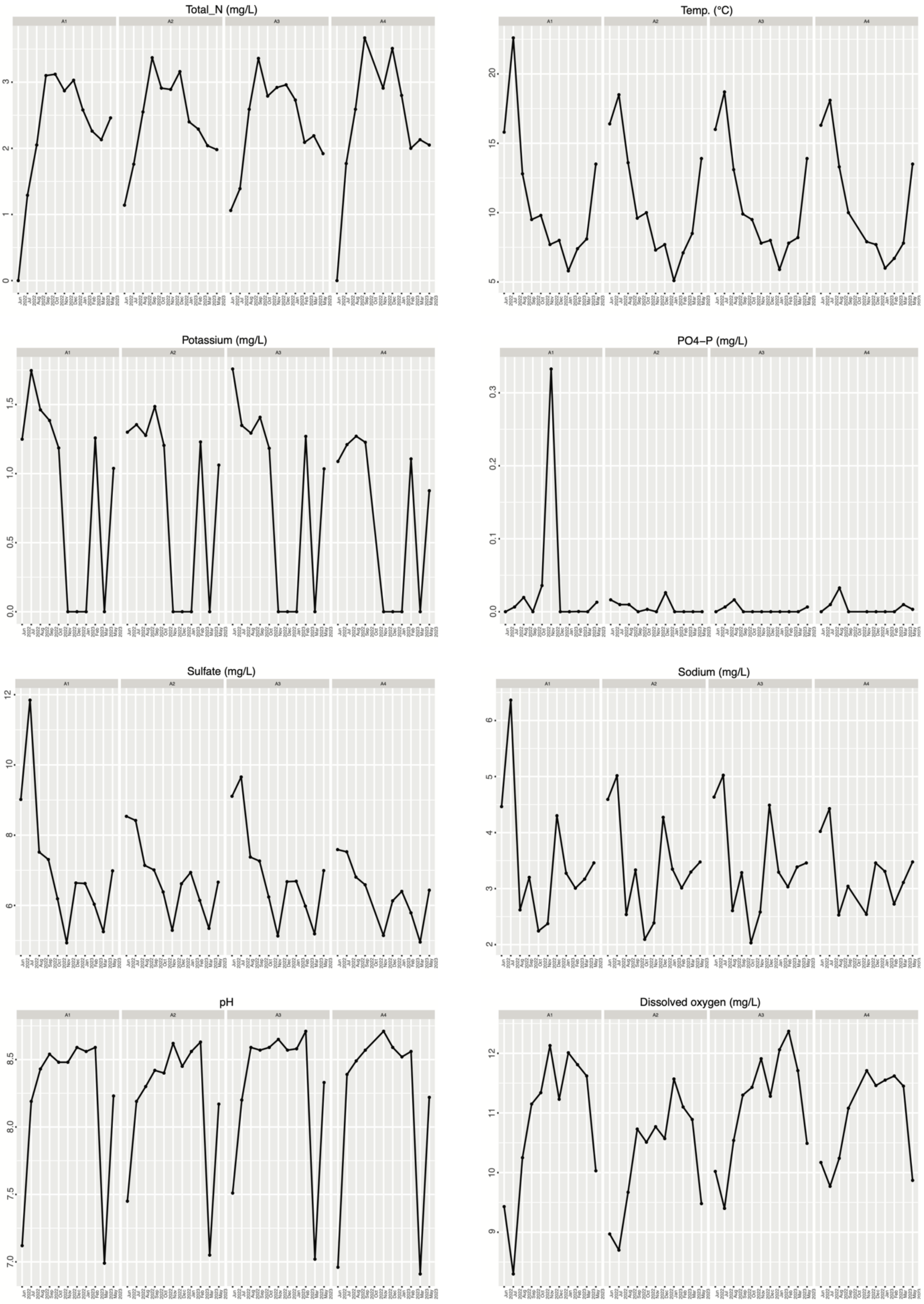
Total_N (mg/L)
Temp. (°C)
Potassium (mg/L)
PO4−P (mg/L)
Sulfate (mg/L)
Sodium (mg/L)
pH
Dissolved oxygen (mg/L)
A1
A2
A3
A4

Fig. S2: Correlation matrix of the different environmental parameters. Correlation values are described in each matrix-cell. Higher to lower correlations are colored from purple to blue, respectively. Significant correlations are also described in the matrix-cells. Significancy codes: **** < 0.0001, *** < 0.001, ** < 0.01, * < 0.05. Empty cells are non-significant correlations.

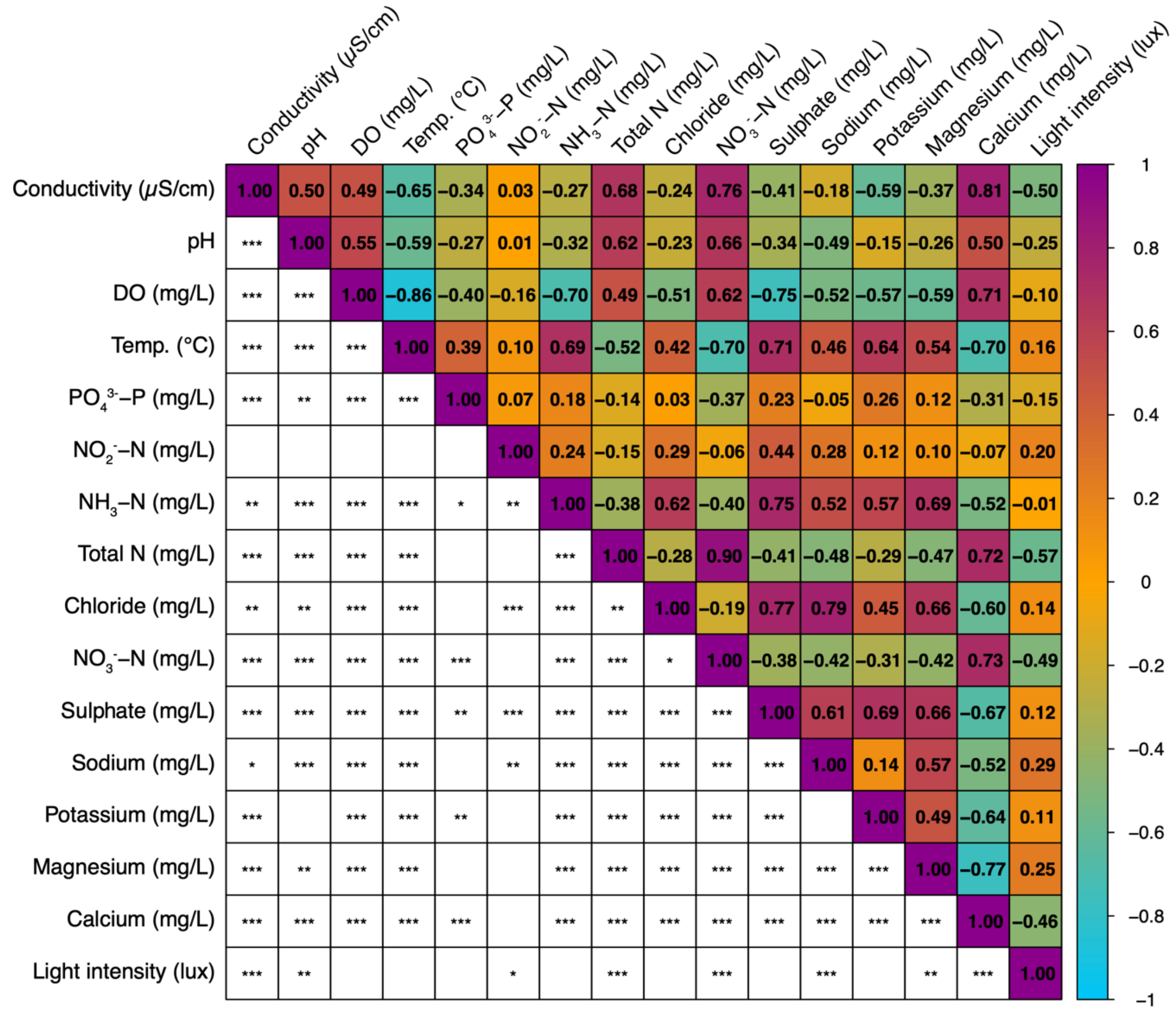

Fig. S3: Principal component analysis (PCA) of all (A) and the selected (B) environmental parameters. Dimensions explain the proportion (%) of the total variance. Variables are colored by their cos2 values, illustrating the quality of representation of the variables on both dimensions.

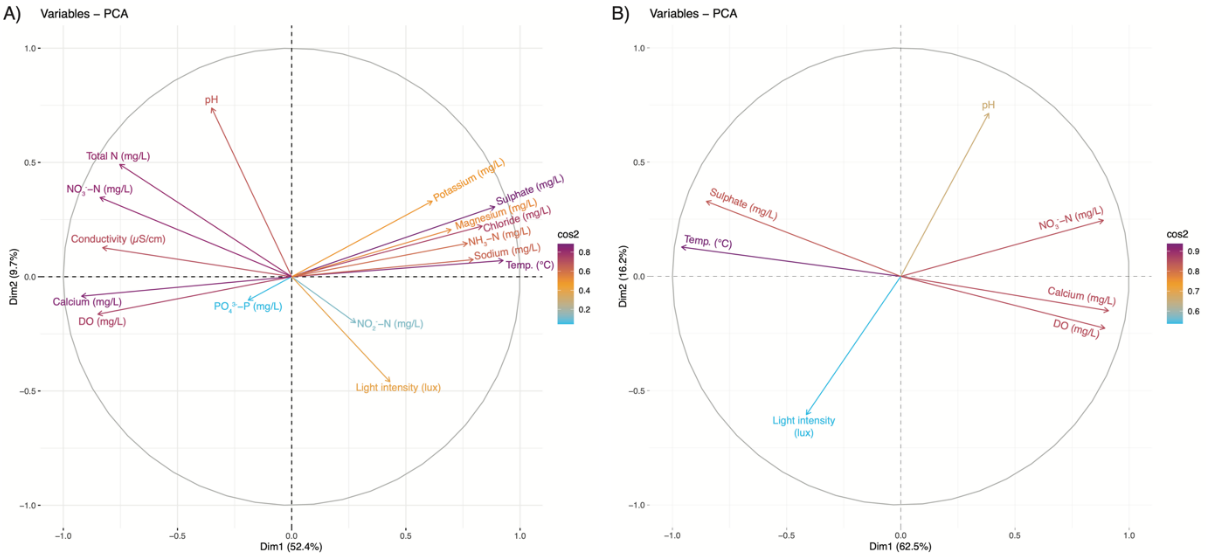

Fig. S4: Diversity analysis of the microbial communities between sequencing batches. (A-C) describe the bacterial communities; (D-F) describe the eukaryotic communities. (A, D) α-diversity of the of the bacterial and eukaryotic communities between sequencing batches calculated with the Shannon index. Boxplots illustrate the interquartile range (IQR); middle line represent the median; the whiskers represent the most extreme data points within 1.5 times the IQR; and the points represent the outliers. Significancy codes: **** < 0.0001, *** < 0.001, ** < 0.01, * < 0.05. (B, E) β-diversity: PCoA of the bacterial and eukaryotic communities calculated using a UniFrac distance metric, respectively. Points represent the samples and dashed ellipses (confidence intervals at 95%). Points with black borders represent the centroids. (C, F) Multivariate dispersions across all sites. Black points represent the means between each sequencing batch.

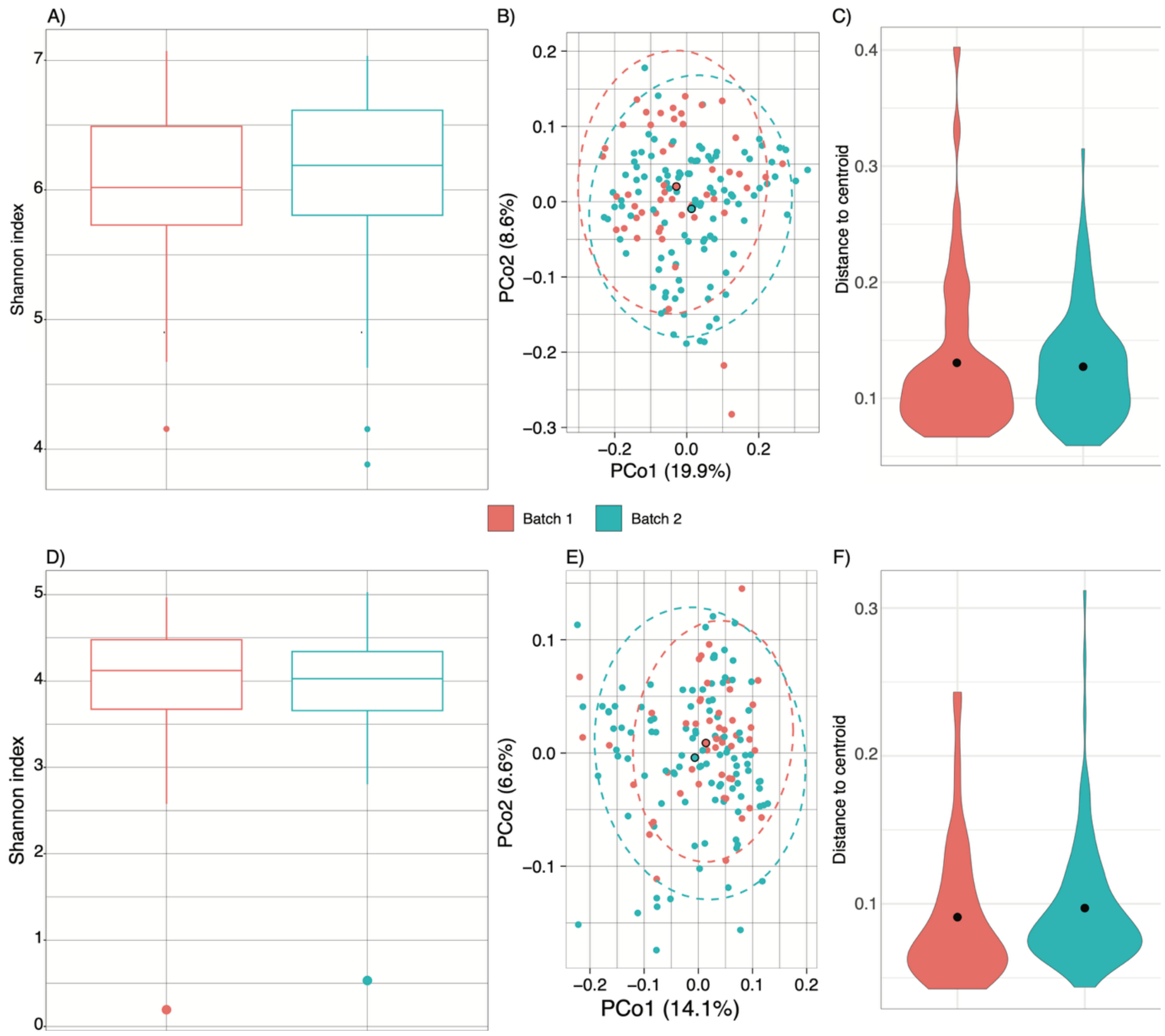

Fig. S5: Diversity analysis of the microbial communities throughout time. (A-C) describe the bacterial communities; (D-F) describe the eukaryotic communities. (A, D) α-diversity of the microbial communities throughout time calculated with the Shannon index. Boxplots illustrate the interquartile range (IQR); middle line represent the median; the whiskers represent the most extreme data points within 1.5 times the IQR; and the points represent the outliers. Significancy codes: **** < 0.0001, *** < 0.001, ** < 0.01, * < 0.05. (B, E) β-diversity: PCoA of the bacterial and eukaryotic communities calculated using a UniFrac distance metric, respectively. Points represent the samples and dashed ellipses (confidence intervals at 95%). Points with black borders represent the centroids. (C, F) Multivariate dispersions throughout time. Black points represent the means between each sampling timepoint.

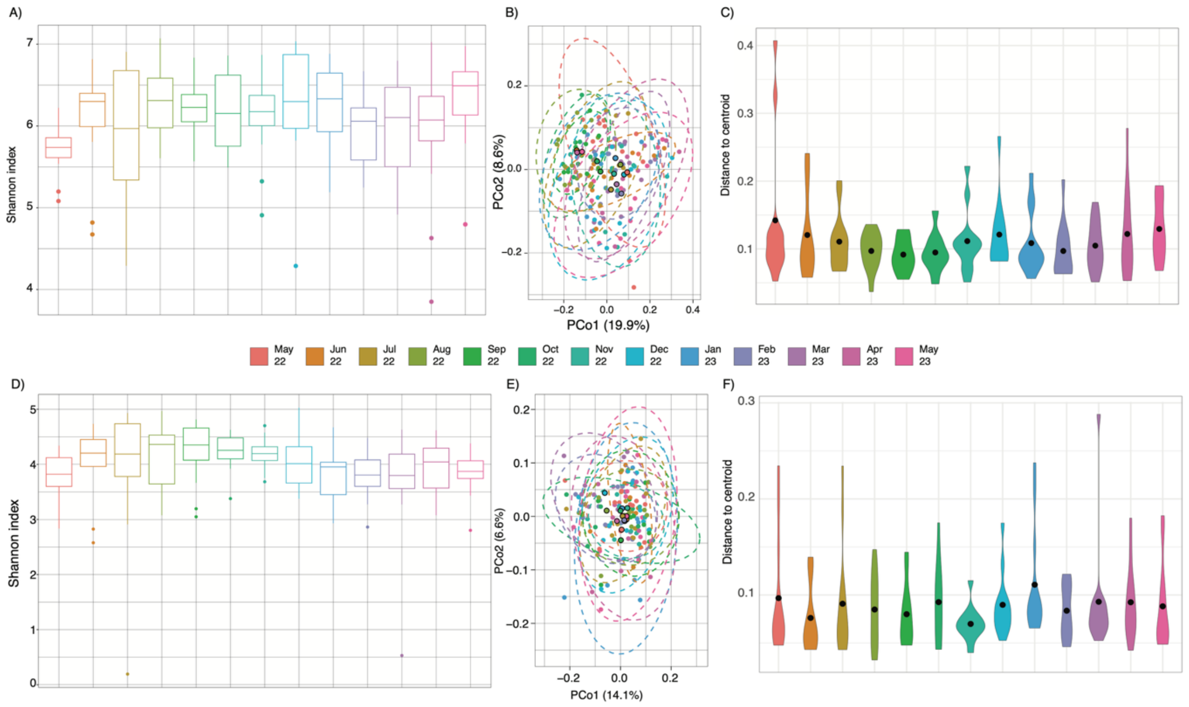

Fig. S6: Temporal and spatial dynamic of the relative abundance of the top 5 phyla of bacteria across all sites (y-axis). The temporal scale is given on the x-axis. Each phylum is separated by color. All other phyla that are not described are included in others (grey bars). Empty bars are samples that could not have been collected due to weather conditions.

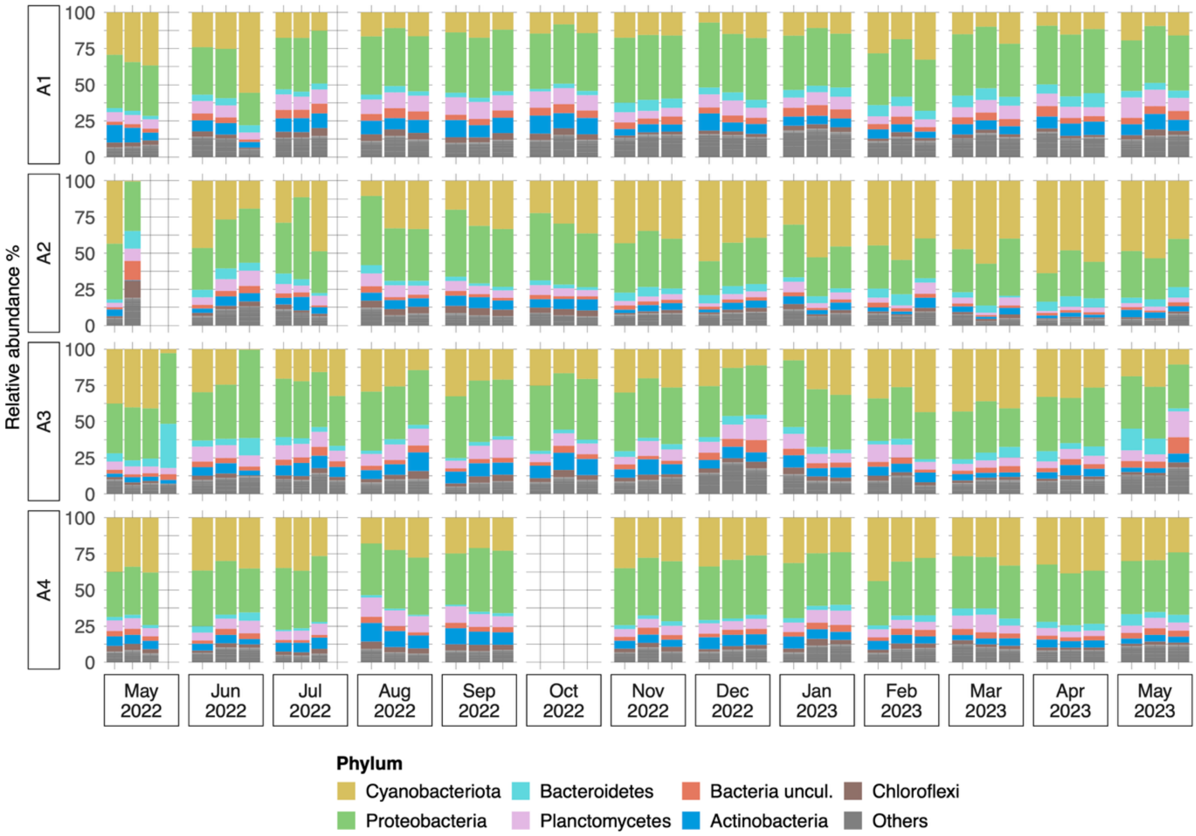

Fig. S7: Temporal and spatial dynamic of the relative abundance of the top 20 genera of eukaryotes across all sites (y-axis). The temporal scale is given on the x-axis. Each genus is separated by color. All other genera that are not described are included in others (grey bars). Empty bars are samples that could not have been collected due to weather conditions.

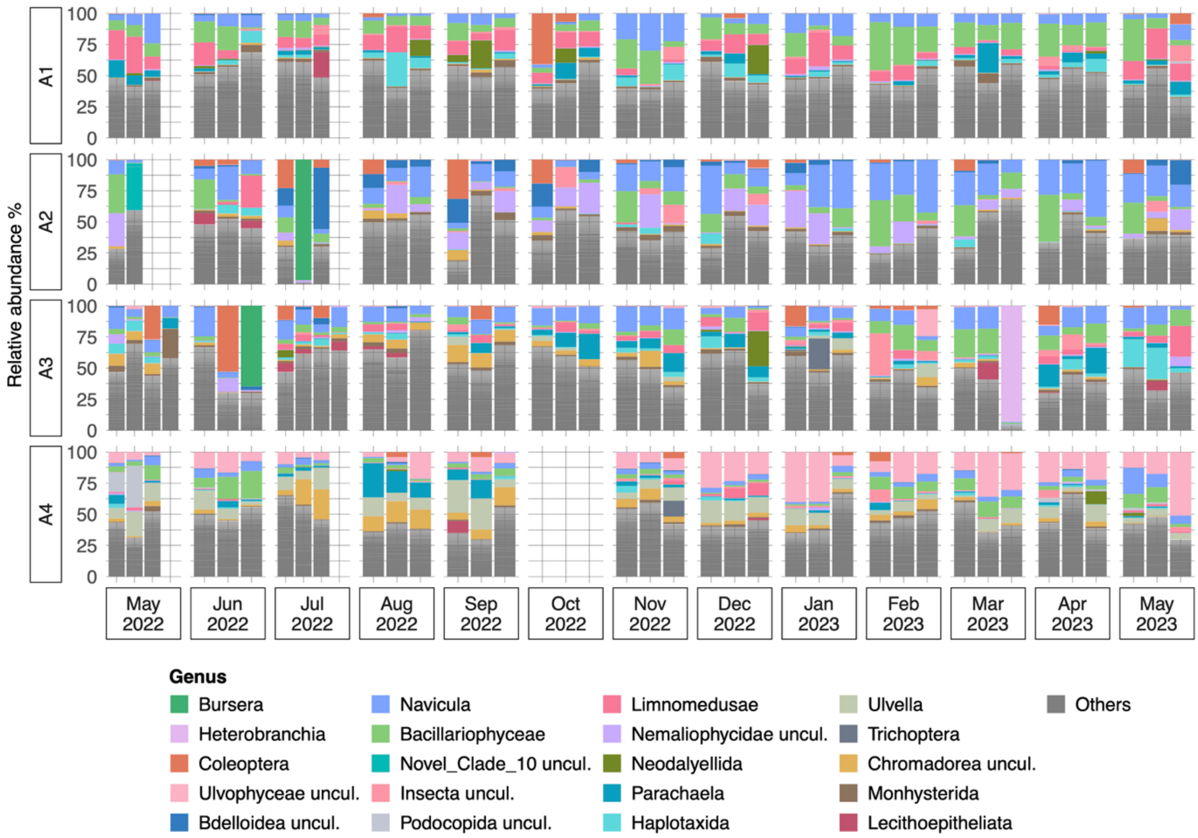

Fig. S8: Co-occurrence network analyses of the microbial communities between each site (minimum relative abundance at 1%). Positive and negative correlations are colored in blue and red, respectively. Correlation strengthens is illustrated by the thickness of the correlation vertices (correlations illustrated: $p < 0.001$ and $r > 0.6$). Nodes are colored by phylum. Bacteria and eukaryotes are differentiated by round and triangle nodes, respectively. No correlations were observed with *Microcoleus* in site A3.

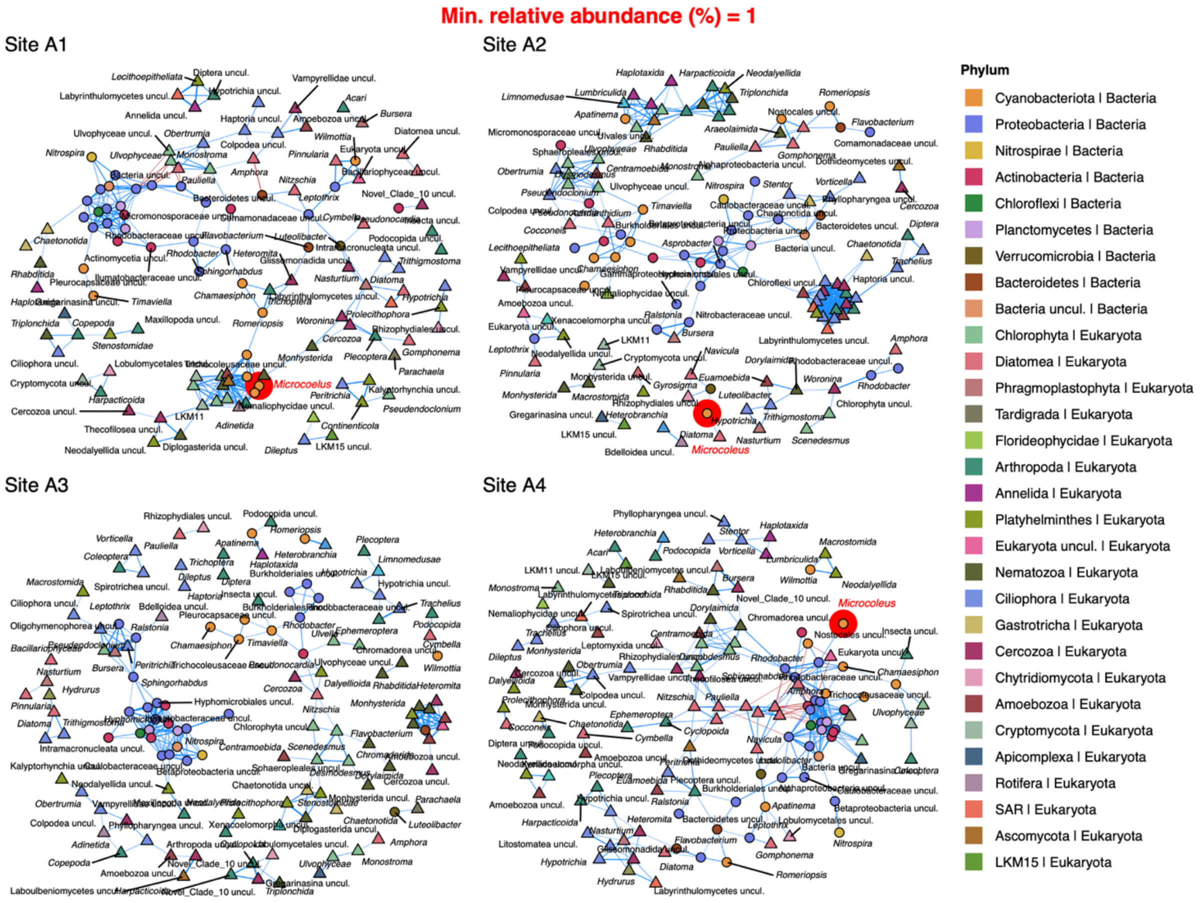

Fig. S9: (A) Water discharge (m3/sec) in the Areuse river between 2022 and 2024. The zone highlighted in blue illustrates the sampling period. (B) Water discharge (m3/sec) in the Areuse river during sampling period. Red vertical dashed lines represent the sampling timepoints.

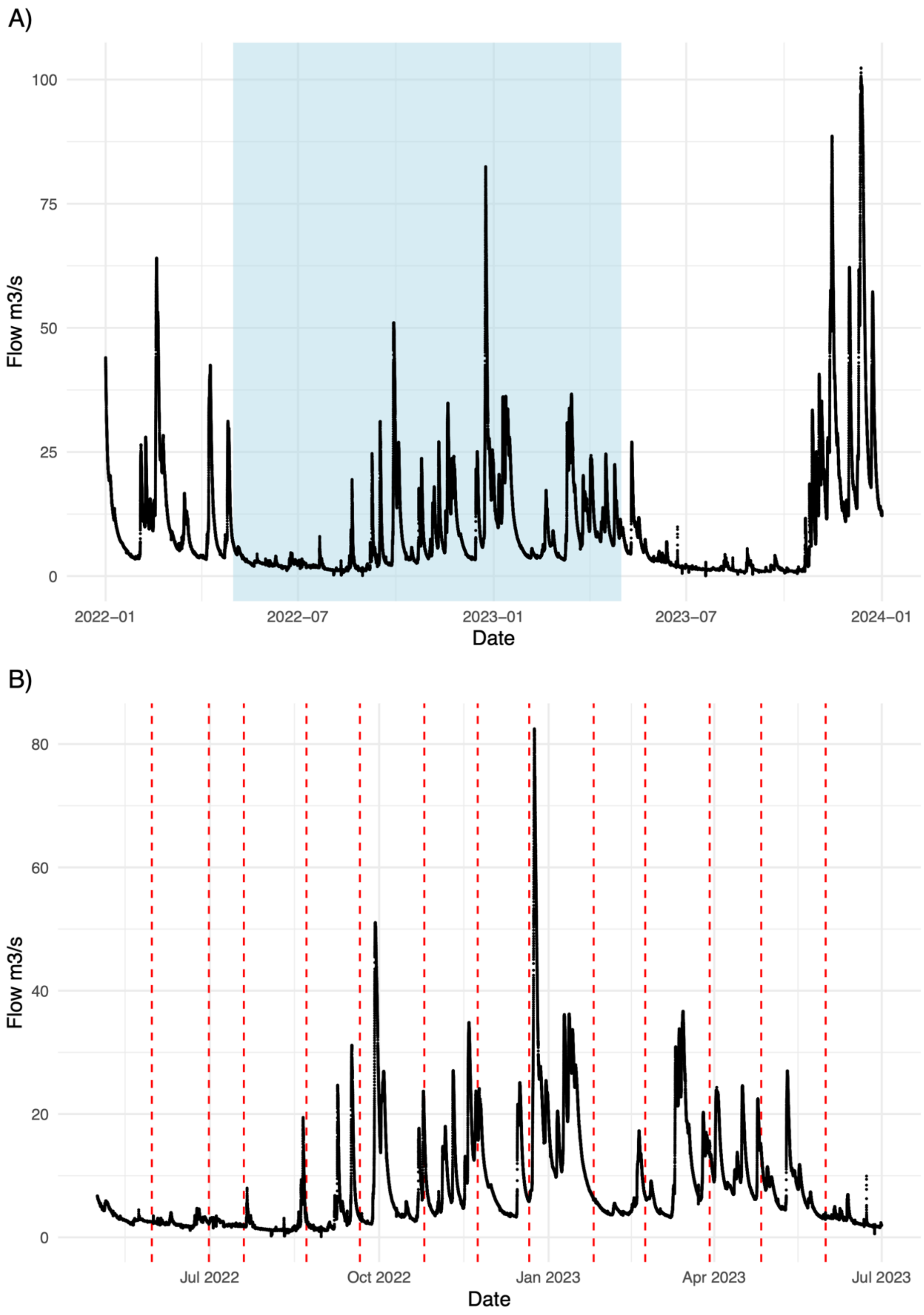

Supplementary Table legends

Table S1: Summary of the bacterial OTUs. Each phylum is described with the number of OTUs, the number of reads, and the number of taxonomic ranks assigned to each phylum.

Table S2: Summary of the eukaryotic OTUs. Each phylum is described with the number of OTUs, the number of reads, and the number of taxonomic ranks assigned to each phylum.

Table S3: Summary of the number reads and OTUs for bacteria, cyanobacteria, and eukaryotes within each sample.

Table S4: Tukey's post-hoc tests between all factor levels. Each factor and levels are described with the p-values of the post-hoc tests. P-values for bacteria and eukaryotes are described in individual columns. Significant p-values (< 0.05) are highlighted in red.

Table S5: Edges (correlations) of the microbial network analysis while filtering the taxa with maximum relative abundances > 2%.

Table S6: Edges (correlations) of the microbial network analysis while filtering the taxa with maximum relative abundances > 1%.

Supplementary information

Supplementary text: Protocol for urea detection in water samples. The protocol was designed by the Plateforme Neuchâteloise de Chimie Analytique at the University of Neuchâtel.

The analysis of urea was performed by ultra-high performance chromatography coupled with tandem mass spectrometry (UHPLC-MS/MS). The system, entirely controlled by Masslynx 4.2, was composed of an Acquity I-Class (Waters) equipped with an FTN autosampler and interfaced with a TQ-XS mass spectrometer (Waters) via an electrospray source. Aqueous samples were diluted 10-fold with acetonitrile and an internal standard of 15N2-urea (Sigma-Aldrich) was added at a concentration of 20 ng/mL. The mixture was placed in TrueView pH control glass vials (Waters) and 3 µL was injected onto an Acquity UPLC BEH HILIC column (2.1x100 mm, 1.7 µm, Waters). The mobiles phases were H2O containing 0.05% formic acid and 1 mM ammonium formate (phase A) and acetonitrile containing 0.05% formic acid (phase B). A gradient starting at 95% B and decreasing at 79.3% B in 2 min, then 50% B in 0.5 min, holding at 50% B for 2.0, followed by reequilibration at 95% B for 3.3 min was applied. The flow rate was 0.4 mL/min. The column temperature was 25°C. Under these conditions, urea and its internal standard eluted at 1.38 min. The mass spectrometer was operated in electrospray positive ionization, using a desolvation temperature of 600°C, a desolvation gas flow of 1000 L/h, a cone gas flow of 250 L/h, and a capillary voltage of 2 kV. The multiple reaction monitoring (MRM) mode was employed using the transitions m/z 61/44 and m/z 63/45 for urea and 15N2-urea, respectively. We verified that no-cross-talk existed between both transitions. A calibration curve with calibration points at 5, 10, 20, 40, 80 and 160 ng/mL, all containing the internal standard at 20 ng/mL, was prepared in 90% acetonitrile. Data processing was performed with TargetLynx XS (Waters). The instrumental limits of detection and quantification were 2 and 5 ng/mL, respectively, corresponding to 20 and 50 ng/mL after sample dilution with acetonitrile.
